# Gene therapy using the human telomerase catalytic subunit gene promoter enables targeting of the therapeutic effects of vesicular stomatitis virus matrix protein against human lung adenocarcinoma

**DOI:** 10.3892/etm.2012.679

**Published:** 2012-08-23

**Authors:** PING ZHANG, JIAO TAN, DA-BING YANG, ZI-CHAO LUO, SHAN LUO, PING CHEN, PING SUN, YI ZHOU, XIAN-CHENG CHEN, YU-QUAN WEI, YAN-JUN WEN

**Affiliations:** 1 State Key Laboratory of Biotherapy and Cancer Center, West China Hospital, West China Medical School, Sichuan University, Chengdu, Sichuan 610041;; 2 CAS/CUHK Research Centre for Biosensors and Medical Instruments, Institute of Biomedical and Health Engineering, Shenzhen Institutes of Advanced Technology, Chinese Academy of Sciences, Shenzhen 518005, P.R. China

**Keywords:** vesicular stomatitis virus matrix protein, phTERT-M, pVAX-M, apoptosis, targeted antitumor effect

## Abstract

The catalytic subunit of telomerase, human telomerase reverse transcriptase (hTERT), is highly active in immortalized cells and more than 90% of human cancer cells, but is quiescent in the majority of normal somatic cells. Thus, the hTERT promoter has been extensively used in targeted cancer gene therapy. Vesicular stomatitis virus (VSV) matrix protein (MP) induces the apoptosis of tumor cells in the absence of other viral components. In our previous studies, we successfully constructed the pVAX-M plasmid from the pVAX plasmid, which expressed wild-type VSV MP (VSV MP is under the control of the CMV promoter) and demonstrated that pVAX-M efficiently suppresses the growth of malignant tumors via the induction of apoptosis *in vitro* and *in vivo*. The present study was designed to construct the plasmid phTERTM (VSV MP is under the control of the hTERT promoter) and investigate whether it had a targeted antitumor effect in nude mice bearing human lung adenocarcinoma. *In vitro*, A549 human lung adenocarcinoma cells were treated with NS, Lip-null, etoposide, Lip-pVAX-M or Lip-phTERT-M, and examined for cell viability through MTT assays or for apoptosis by flow cytometry and TUNEL assays. *In vivo*, A549 human lung carcinoma models in nude mice were established. Mice were treated with 10 4-weekly intravenous administrations of NS, Lip-null, etoposide (2 mg/kg), Lip-pVAX-M or Lip-phTERT-M. Subsequently, Lip-phTERT-M was found to be the most efficient inhibitor of tumor growth and inducer of tumor cell apoptosis when compared with the other groups *in vivo* and *in vitro* (P<0.05). Notably, immunohistochemical staining showed that Lip-phTERT-M significantly limited the overexpression of VSV MP to the tumor tissues and reduced VSV MP expression in other organs in comparison with Lip-pVAX-M (P<0.05). Therefore, it can be concluded that phTERT-M demonstrates a targeted antitumor effect on A549 human lung adenocarcinoma cells. These observations suggest that phTERT-M gene therapy may be a novel and potent strategy for targeting human lung adenocarcinoma.

## Introduction

Human telomerase is a specialized DNA polymerase which controls the replication of chromosomal ends, or telomeres. The majority of malignant tumors express telomerase but most normal cells do not (1,2). Therefore, telomerase may be a good candidate for targeted cancer gene therapy. Human telomerase consists of 3 major components: the RNA component (hTER); the telomerase-associated protein (hTEP1); and the telomerase catalytic unit or human telomerase reverse transcriptase (hTERT) (3–5). Both hTER and hTERT are necessary for telomerase activity, although telomerase expression is predominantly regulated at the transcriptional level of hTERT. Additionally, hTERT expression is specific to human tumor cells, whereas hTER is present in normal and tumor cells. Thus, the hTERT promoter has been extensively used in targeted cancer gene therapy (6–13).

Vesicular stomatitis virus (VSV) is a negative-stranded RNA rhabdovirus with a single molecule genome. VSV includes 5 major proteins [nucleoprotein (N); phosphoprotein (P); matrixprotein (M); glycoprotein (G) and polymerase (L)] and selectively replicates in interferon (IFN)-resistant tumor cells. VSV also induces host cell apoptosis via signaling through the double-stranded RNA-dependent serine/threo-nine protein kinases, Fas and Daxx (14–16). Studies have confirmed that VSV efficiently suppresses the growth of syngeneic tumors in immunocompetent mice and human tumor xenografts in nude mice and prolongs the survival time of tumor-bearing animals. However, severe adverse effects, including flu-like symptoms, encephalitis, ventriculitis, oral vesicles and cervical lymphadenopathy, limit the clinical applications of replication-competent VSV (17–20). Matrix protein (MP) is important in viral assembly and cytopathogenesis. Expression of MP alone causes a number of the same cellular effects as infection with VSV via the inhibition of host gene transcription and nucleocytoplasmic transport of host RNAs and proteins (21–24). Previously, we constructed the plasmid pVAX-M (VSV MP is under the control of the CMV promoter) and revealed that pVAX-M alone or combined with radiation or DDP efficiently inhibits solid tumor growth and significantly prolongs survival (25–29). These findings suggest that VSV MP is a promising agent for the treatment of tumors.

In the present study, in order to realize the targeted anti-tumor effect of VSV MP, the plasmid phTERT-M (VSV MP controlled by the hTERT promoter) was constructed. Subsequently, we found that phTERT-M suppressed the tumor growth of the A549 model, limited VSV MP overexpression to the tumor tissues and reduced VSV MP expression in other organs more effectively than pVAX-M. These results demonstrate that phTERT-M gene therapy is a more specific and safer approach for the treament of human lung adenocarcinoma than pVAX-M gene therapy.

## Materials and methods

### Cell line

The human lung adenocarcinoma A549 cell line was obtained from the American Type Culture Collection (ATCC, Rockville, MD, USA). It was cultured in RPMI-1640 medium supplemented with 10% heat-inactivated fetal bovine serum (FBS), 100 U/ml of penicillin, 100 mg/ml of streptomycin and maintained in a 37°C incubator with a humidified 5% CO_2_ atmosphere.

### Plasmid construction, preparation of cationic liposome and liposome-DNA complex

pVAX-M from the pVAX plasmid (Invitrogen Life Technologies, San Diego, CA, USA) expressing wild-type VSV MP, was constructed in our laboratory previously (25–29). As a control, pure pVAX plasmid without VSV MP-cDNA was used as an empty vector (null).

A 271-bp fragment containing the core promoter region essential for the transactivation of hTERT was synthesized and subcloned into the *Bgl*I*/Hin*dIII-digested pVAX-MP plasmid to produce the phTERT-M plasmid (29,30). The recombinant plasmid, phTERT-M, was confirmed to contain the correct sequence by nucleotide sequencing.

The procedure for preparing plasmid and liposome was performed as described in our previous studies (25–29). DNA-liposome mixtures were prepared 30 min prior to use. DNA and stock liposome were diluted in 5% dextrose in water (D5W) or RPMI-1640 medium without serum and mixed in equal volumes, with a DNA/liposome ratio of 1:3 (μg/μg). All reagents were diluted and mixed at room temperature.

### Cell viability assay in vitro

The cytotoxicity of phTERT-M-liposome mixtures or pVAX-M-liposome mixtures on A549 cells was determined using the 3-(4,5-dimethylthiazol-2-yl)-2,5-diphenyl tetrazolium bromide (MTT; Sigma, St. Louis, MO, USA) colorimetric assay. Cells (∼5,000 cells/100 μl medium) were seeded in each well of a 96-well plate and incubated overnight. The cells were then treated with NS, Lip-null (0.2 μg pVAX/0.6 μg liposome mixtures), Lip-phTERT-M (0.2 μg phTERT-M/0.6 μg liposome mixtures), Lip-pVAX-M (0.2 μg pVAX-M/0.6 μg liposome mixtures) and etoposide (0.1 μg/ml), respectively. Six wells were included in each group. After a 48-h incubation, the medium was aspirated and 20 μl of 5 mg/ml MTT was added per well and incubated at 37°C for 4 h; then supernatant fluid was removed and 150 μl dimethyl sulfoxide (DMSO) was added per well. Spectrometric absorbance at 540 nm was measured using a microplate reader. The cell survival rate was assessed as percent cell viability in terms of non-treated control cells.

### Assessment of apoptosis in vitro

Cell apoptosis was evaluated by flow cytometry and TUNEL assay (DeadEnd Fluorometric TUNEL System; Promega Corporation, Madison, WI, USA). Flow cytometric analysis was performed as previously reported (26,27). Cells (∼2x10^5^ cells/well) were plated in 6-well plates and treated with NS, Lip-null (2 μg pVAX/6 μg liposome mixtures), Lip-phTERT-M (2 μg phTERTP-M/6 μg lipo-some mixtures), Lip-pVAX-M (2 μg pVAX-M/6 μg liposome mixtures) or etoposide (0.1 μg/ml). After a 48-h incubation, the cells were collected and resuspended in 1 ml hypotonic fluorochrome solution containing 50 μg/ml propidium iodide (PI) in 0.1% sodium citrate with 0.1% Triton X-100 and then analyzed by flow cytometry. Cells appearing in the sub-G1 stage were considered as apoptotic cells. To quantify apoptotic cells within the total tumor cells *in vitro*, TUNEL assays were performed according to the manufacturer’s instructions. Cell nuclei with dark green fluorescent staining were defined as TUNEL-positive nuclei. TUNEL-positive nuclei were monitored by fluorescence microscope (Leica, Bensheim, Germany). Five equal-sized fields at x200 magnification were randomly chosen and analyzed. The apoptotic index (AI) was defined as follows: AI (%) = 100 x (apoptotic cells/total tumor cells).

### Animal studies

Female athymic BALB/c nude mice (SPF grade; 6- to 8-weeks old), were purchased from the Laboratory Animal Center of Sichuan University and allowed to acclimate for 1 week before use. All the animal studies were carried out in accordance with institutional guidelines referring to animal use and care.

A549 cells (∼5×10^5^) were injected into the right flank of each nude mouse via subcutaneous inoculation. When the size of the tumors reached ∼30 mm^3^, mice were randomly assigned into 5 groups and treated with NS (100 μl), Lip-null (10 μg pVAX/30 μg liposome mixtures, 100 μl), Lip-phTERT-M (10 μg pVAX-MP/30 μg liposome mixtures, 100 μl), Lip-pVAX-M (10 μg pVAX-M/30 μg liposome mixtures, 100 μl) or etopo-side (2 mg/kg). The animals received 10 4-weekly intravenous administrations and were monitored every 3 days for tumor burden, cachexia and other abnormalities. Tumor sizes were measured using the formula A x B^2^ x 0.52 (A, length; B, width; all in mm). All data are presented as mean ± SD. Mice were sacrificed by cervical dislocation when the volume of the tumor exceeded 6,000 mm^3^. Tissues of interest, including heart, liver, spleen, lung, kidney and tumor, were excised and fixed in 10% neutral-buffered formalin solution or frozen at −80°C.

### Histological analysis

To analyze the targeted antitumor effect of phTERT-M against the A549 model, immunohistochemical staining was performed as described previously (26). The tissues of each group were embedded in paraffin and cut into 3- to 5-μm sections. These sections were then deparaffinized in xylol and rehydrated through a graded alcohol series. Antigen retrieval was performed by autoclaving sections in 10 mM EDTA (pH 6.0) and incubating them with rabbit immunoserum at 1:50 dilution, followed by an incubation with biotinylated rat anti-rabbit antibody and then streptavidin biotin reagents.

To quantify apoptotic cells within the tumor sections, TUNEL assays (DeadEnd Fluorometric TUNEL System; Promega Corporation) were performed according to the manufacturer’s instructions. Five equal-sized fields at x200 magnification were randomly chosen and analyzed. The apoptotic index (AI) was defined as explained above.

### Toxicity observation

No significant differences in weight were found among the 5 groups. No adverse consequences in other gross measures, including ruffling of fur, behavior, feeding and toxic death, were observed in the Lip-phTERT-M group. Furthermore, no significant differences in liver, lung, kidney, spleen, heart, pancreas or brain were observed by hematoxylin and eosin histological examination between the Lip-phTERT-M and NS groups.

### Statistical analysis

All the data were analyzed by the statistical software SPSS 16.0. Data were assessed by ANOVA and Student’s t-test. P<0.05 was considered to indicate a statistically significant difference.

## Results

### The antitumor efficacy of Lip-phTERT-M on A549 cells in vitro

**Figure 1:**
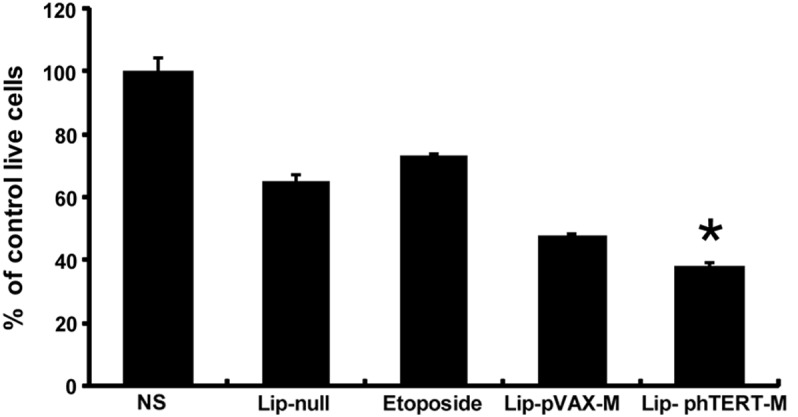
Cell viability assay *in vitro* by 3-(4,5-dimethylthiazol-2-yl)-2,5-diphenyl tetrazolium bromide (MTT). The MTT assay was performed to observe the viability of cells as described in Materials and methods. The proportion of surviving cells was calculated as a percentage of the control. Data are represented as the mean ± SD of 3 independent experiments. The results showed that both Lip-phTERT-M and Lip-pVAX-M suppressed the growth of A549 cells *in vitro*. However, Lip-phTERT-M had a superior inhibitory effect. (^*^P<0.05, compared with the other groups.)

**Figure 2:**
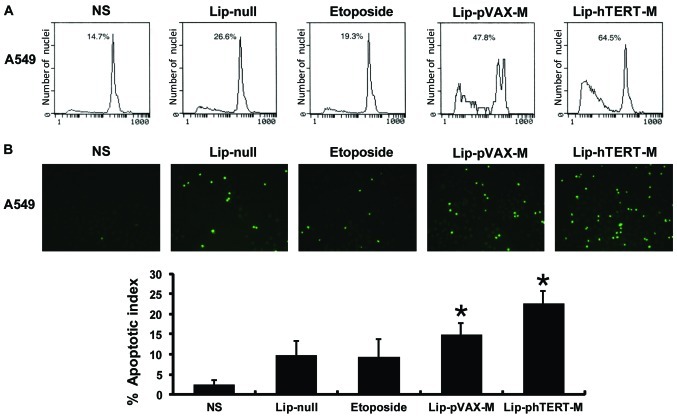
Assessment of apoptosis *in vitro*. The efficacy of inducing A549 cell apoptosis *in vitro* was evaluated by flow cytometry and TUNEL assays. (A) Flow cytometry quantitation revealed that Lip-phTERT-M resulted in the highest apoptotic rate of A549 cells. (B) TUNEL assays showed that Lip-phTERT-M and Lip-pVAX-M increased the apoptotic index (AI) compared with the other groups (^*^P<0.05). However, Lip-phTERT-M resulted in a larger increase in AI compared with Lip-pVAX-M (^*^P<0.05). Data represent the mean AI ± SD of cancer cells.

In order to evaluate the antitumor activity of Lip-phTERT-M on A549 cells *in vitro*, cell viability assays using MTT were performed. The results demonstrated that Lip-phTERT-M reduced A549 cell growth more effectively than the other groups (Fig. 1). The apoptotic effect of the 5 groups on A549 cells was quantitated via flow cytometry and TUNEL assays. From the results of flow cytometry, we revealed that the apoptotic cells accounted for 64.5% of the cells in the Lip-phTERT-M group versus 47.8% in the Lip-pVAX-M group, 26.6% in the Lip-null group and 14.7% in the NS group. Furthermore, the results of the TUNEL assays suggested that the AI had been increased the most by Lip-phTERT-M compared with the other groups. Data are represented as the mean AI ± SD of cancer cells, as percentage normalized to the AI of the cancer cells (Fig. 2).

### Lip-phTERT-M significantly supresses tumor growth in the A549 tumor model

**Figure 3:**
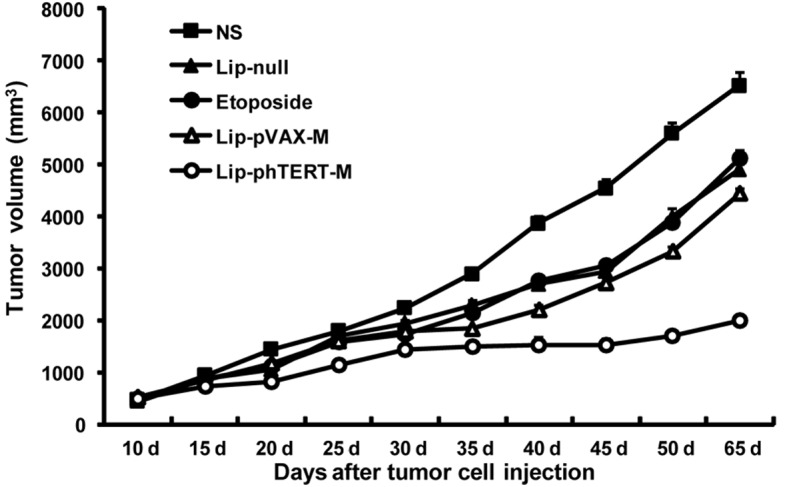
Tumor suppression of the A549 models. In the A549 tumor model, Lip-phTERT-M significantly suppressed tumor growth in comparison with the other groups (^*^P<0.05). Points, average tumor volume; bars, mean ± SD.

The mouse tumor model assay showed that Lip-phTERT-M was more effective in the suppression of tumor growth than the other groups (P<0.05). Additionally, Lip-phTERT-M resulted in >67% inhibition of tumor growth compared with the NS group (P<0.05, 2 days after the completion of treatment). No significant difference in tumor volume was observed among the other groups (Fig. 3).

### Lip-phTERT-M increases intratumoral apoptosis in the A549 tumor model

**Figure 4:**
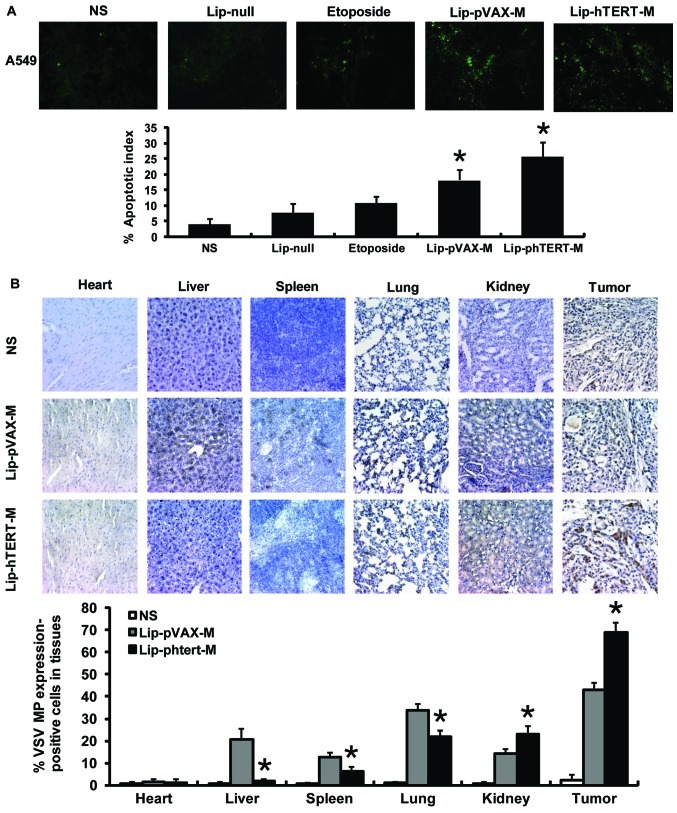
Histochemical staining analysis. (A) Apoptotic cells within tumor tissues were evaluated by TUNEL assays. An apparent increase in the number of apoptotic cells and AI was observed within the tumor tissues in the Lip-phTERT-M and the Lip-pVAX-M groups compared with the other groups (^*^P<0.05). However, Lip-phTERT-M produced a greater increase. Data represent the mean AI ± SD of cancer cells. (B) Targeted antitumor effect analysis by immunohistochemical staining. The results indicated that Lip-phTERT-M restricted VSV MP overexpression to the tumor tissues rather than the other organs and had a superior specific antitumor effect (^*^P<0.05, compared with Lip-pVAX-M). AI, apoptotic index.

The presence of apoptotic cells within the tumor sections was determined by TUNEL assays. The findings demonstrated that Lip-pVAX-M and Lip-phTERT-M enhanced the apoptotic rate of tumor cells, and a more apparent increase in the number of apoptotic cells was observed within the tumors of the Lip-phTERT-M group. Data are represented as the mean AI ± SD of cancer cells, as a percentage normalized to the AI of the cancer cells (Fig. 4A).

### Lip-phTERT-M exhibits a targeted antitumor effect on the A549 tumor model

To investigate the targeted antitumor effect of Lip-phTERT-M, immunohistochemical staining was carried out. The results demonstrated that Lip-phTERT-M resulted in an apparent increase in VSV MP expression in the tumor and the kidney cells, whereas expression in the liver, the spleen and the lung was significantly reduced in comparison with Lip-pVAX-M (P<0.05). Moreover, VSV MP expression in the heart in the two groups was low and there was no significant difference between them. These findings demonstrate that Lip-phTERT-M restricted abundant VSV MP expression to the tumor tissues and may have a superior specific antitumor effect to Lip-pVAX-M (Fig. 4B).

## Discussion

In order to enhance the efficacy and safety of cancer gene therapy, numerous scientific teams focus on restricting the therapeutic gene expression to tumors. If the therapeutic gene is expressed in all cells, it will affect tumor and normal cells. A tumor-specific promoter system is likely to be useful for solving this problem. However, true tumor-specific promoters are rare and often useful only in particular types of cancer (31,32). hTERT is the catalytic subunit of telomerase, which is highly active in immortalized cells and >90% of human cancers but is inactive in most normal somatic cells. It is apparently a strong and tumor-selective promoter with potential applications in targeted cancer gene therapy (31–33).

VSV MP induces the apoptosis of tumor cells in the absence of other viral components without severe side-effects, unlike VSV (21–24). Our previous studies demonstrated that the plasmid pVAX-M alone or combined with radiation or DDP efficiently inhibited the growth of solid tumors and significantly prolonged survival times (25–29). Thus, VSV MP gene therapy is a promising approach for the treatment of tumors.

In the present study, the plasmid phTERT-M was constructed to investigate whether it was able to inhibit tumor growth selectively and specifically. We discovered that Lip-phTERT-M suppressed A549 cells or the tumor model growth more effectively by inducing a higher rate of apoptosis in A549 cells than Lip-pVAX-M *in vitro* and *in vivo*. These findings suggest that Lip-phTERT-M had an enhanced anti-tumor efficacy against A549 human lung adenocarcinoma cells compared with Lip-pVAX-M. Secondly, Lip-phTERT-M resulted in an apparent increase in VSV MP expression in the tumor and kidney, whereas its expression in the liver, the spleen and the lung were significantly reduced in comparison with Lip-pVAX-M (P<0.05). These findings may further demonstrate that Lip-phTERT-M limited the overexpression of VSV MP to the tumor tissues and had a stronger targeted antitumor effect on A549 models in comparison with Lip-pVAX-M. Additionally, Lip-pVAX-M causes more abundant VSV MP expression in the tumors than in the organs, thereby improving its antitumor efficacy and safety. Thus, it may be proposed that the enhanced antitumor efficacy of Lip-phTERT-M is strongly correlated with its targeted antitumor effect.

Our data demonstrated that phTERT-M gene therapy had an apparent targeted antitumor effect against A549 human lung carcinoma models. Given the strong antitumor effect and minimal toxicity, the results of our study may be of significance to the further exploration of the potential applications of this approach in the treatment of human lung adenocarcinoma.
